# Oxidative Stress and Survival of *Leishmania* spp.: A Relationship of Inverse Proportionality for Disease Outcome

**DOI:** 10.1017/erm.2025.10010

**Published:** 2025-06-20

**Authors:** Souravi Roy, Mayumi Mandal, Moumita Halder, Pijush K. Das, Anindita Ukil

**Affiliations:** 1 Department of Biochemistry, University of Calcutta, Kolkata, India; 2 Infectious Diseases and Immunology Division, CSIR – Indian Institute of Chemical Biology, Kolkata, India

**Keywords:** leishmaniasis, macrophages, oxidative stress, reactive oxygen species (ROS), signalling pathways

## Abstract

The development of oxidative stress depends on the deregulation of the fine balance between the proportion of reactive oxygen species (ROS) generation and the activity of antioxidants. As oxidative stress is deleterious for *Leishmania* sp., their host cell generates ROS as one of the foremost defence strategies. The parasites have also raised a variety of counteracting tricks in order to conquer this challenge. Upon infection, the host’s own antioxidant system is activated by the parasite to neutralise the oxidative stress-mediated protection. In addition to using the host’s antioxidant mechanisms, some genes within the parasite also make them more tolerant against oxidative stress. Therefore, the present review focuses on some major regulators intimately related to the equilibrium between oxidation and antioxidation following infection with *Leishmania* sp., which may be helpful in developing a comprehensive knowledge of this specific wing of infection biology associated with oxidative stress.

**Search Results:**

Reactive oxygen species (ROS) play a dual role in leishmaniasis by contributing to both host defence and parasite survival mechanisms. In the host, ROS promote parasite clearance through induction of apoptosis, activation of pro-inflammatory signalling pathways (e.g., MAPK, JNK), inflammasome assembly, and M1 macrophage polarisation. Conversely, Leishmania species have evolved multiple strategies to neutralize ROS, including the upregulation of host antioxidant enzymes like HO-1, inhibition of ROS-producing pathways, and expression of parasite-derived antioxidants such as SOD, GPx, and trypanothione reductase. The parasite alsoadapts through gene regulation and metabolic changes to counter oxidative stress. Importantly, ROS have emerged as key targets for antileishmanial therapies, with various drugs and natural compounds shown to induce ROS-mediated parasite death, highlighting their potential in future therapeutic development.

**Conclusions:**

In summary, the survival of Leishmania hinges on its ability to counteract host-induced oxidative stress. Targeting its antioxidant defences and enhancing host ROS production can disrupt this balance, leading to parasite death. Exploring ROS-related signalling offers a promising path for developing effective therapies against leishmaniasis.

## Introduction

**Figure 1.**
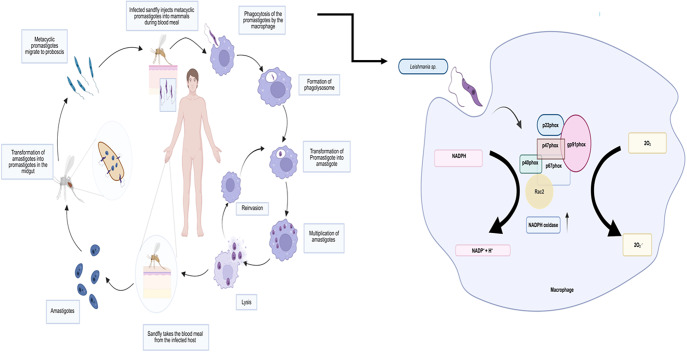
Life cycle of *Leishmania* sp. and the process of ROS generation in macrophages during infection (Ref. 4).

Leishmaniasis is a vector-borne disease caused by the obligate intracellular parasite *Leishmania* sp. The disease can be classified into four main categories depending on the species of *Leishmania* involved. These are cutaneous, mucocutaneous, diffused cutaneous and visceral leishmaniasis. Cutaneous leishmaniasis (CL) is mainly caused by *Leishmania major, Leishmania mexicana*, and *Leishmania tropica* and typically occurs at the site of sand fly bite as a solitary non-suppurative papule. Mucocutaneous leishmaniasis is mainly caused by *Leishmania braziliensis and Leishmania panamensis* (Ref. 1), and it is the most disfiguring form of the disease, which leads to facial deformities. Diffused cutaneous leishmaniasis (DCL), caused by *Leishmania amazonensis* and *Leishmania pafinoi*, is a rare anergic form of CL characterised by widespread lesions resembling lepromatous leprosy. It starts with a primary lesion and spreads due to impaired cellular immunity. DCL is often observed with multiple (>10) lesions, including papules, nodules or ulcers, and responds well to standard treatment (Ref. 2). The most lethal form of leishmaniasis is Visceral Leishmaniasis (VL), which is also known as kala-azar. In this case, a systemic infection occurs, affecting visceral organs such as the liver and spleen, and is fatal if untreated. The species that are associated with VL include *Leishmania donovani*, *Leishmania infantum* and *Leishmania chagasi* (Ref. 3). There are two distinct phases in the life cycle of *Leishmania* sp.: promastigote and amastigote phases (Figure 1). Female sand flies (*Phlebotomus* and *Lutzomyia* species) acquire *Leishmania* parasites while feeding on an infected host. Amastigotes, found in skin macrophages, are released due to tissue damage caused by the fly’s saw-like mouthparts. Once ingested, the parasites experience temperature and pH changes in the sand fly midgut, triggering their transformation into flagellated promastigotes. Initially, pro-cyclic promastigotes multiply within the peritrophic matrix before differentiating into highly motile nectomonad promastigotes, which migrate and attach to the midgut epithelium. They then transform into metacyclic promastigotes, that is, the infective stage. The *Leishmania* life cycle begins when an infected female sand fly injects metacyclic promastigotes into the host while feeding. These promastigotes are quickly engulfed by phagocytic cells, such as macrophages and neutrophils. Due to the short lifespan of neutrophils, macrophages serve as the primary host cells where the parasite proliferates. Once inside the macrophage’s phagolysosome, the promastigotes transform into non-motile amastigotes, which multiply through binary fission. As they continue to replicate, the macrophage eventually ruptures, releasing the parasites to infect nearby cells. After infection, both neutrophils and macrophages are drawn to the site, playing a crucial role in disease progression. Studies indicate that neutrophils, being highly efficient at parasite uptake, act as short-lived intermediate hosts. They function as ‘Trojan horses’, allowing *Leishmania* to enter macrophages unnoticed, preventing immune activation. *L. major* infection delays neutrophil death and promotes MIP-1β secretion, which attracts macrophages. These macrophages then engulf free parasites and infected or apoptotic neutrophils, becoming the primary host cells for parasite replication and the key effectors in parasite elimination (Ref. 4).

Interestingly, the primary mammalian host of *Leishmania* is macrophages, which are well equipped with an elaborate defence mechanism (Ref. 5), and the first line of defence put forward by macrophages is the generation of reactive oxygen species (ROS) (Ref. 6). The enzyme primarily responsible for ROS production is multisubunit NAD(P)H-dependent phagocytic oxidase (Phox or NOX2), expressed in professional phagocytic cells such as neutrophils and monocytes/macrophages (Ref. 7). Upon sensing foreign particle, subunits of this enzyme complex get assembled and become activated, thereby resulting in the generation of ROS. Apart from Phox, several other mechanisms further contribute to ROS generation, and neutralisation of this oxidative burst is crucial for successful intracellular survival of pathogens. In order to implement this, the parasite takes various counteracting tricks (Ref. 8). To understand the detailed mechanism of host–pathogen interaction in the context of leishmaniasis, it is necessary to evaluate the oxidative stress-dependent defence of host macrophage and its counteraction by the pathogen. In the present review, we have tried to discuss all the pathways adopted by the host and their possible neutralisation by *Leishmania* for the successful establishment of infection.

We have compiled this review article to explore the impact of oxidative stress on the survival of *Leishmania* species. For our analysis, we focused on publications from 2009 onwards, selecting studies based on a targeted search strategy. In total, we found 101 papers that directly addressed these themes. Additionally, a few more studies were included after further searching for research that examined the role of oxidative stress in other pathogens, as these provided valuable insights for a more comprehensive understanding of the subject. Among the 101 resulting papers, only those that focused on different *Leishmania* species were included, as studies that dealt with general aspects of the disease were excluded from the analysis. The chosen papers specifically address topics related to oxidative stress, *Leishmania* species and parasite survival, ensuring a relevant and focused review.

## Generation of host oxidative burst and its effect on *Leishmania* infection

At the time of phagocytosis of *L. donovani* promastigotes, ROS is generated by the host macrophage to kill the pathogens (Ref. 6). In addition to macrophages, several types of white blood cells are also associated with the induction of ROS during VL. In this regard, eosinophils have a protective role as these cells are associated with the production of ROS following *L. infantum* infection, which eventually results in reduced parasitic load (Ref. 9). Beside this, neutrophils are also associated with ROS generation during the course of *L. infantum* infection (Ref. 10). Different types of ROS are associated with different forms of leishmaniasis. In VL, the immune system generates a variety of ROS as part of the oxidative burst to combat the infection. The superoxide anion is produced by the enzyme Nicotinamide Adenine Dinucleotide Phosphate (NADPH) oxidase during the oxidative burst, which is activated in macrophages and neutrophils in response to infection. Mice deficient in NADPH oxidase exhibit impaired parasite clearance, while excessive ROS can cause tissue damage. Targeting the oxidative stress response in *Leishmania* or enhancing ROS production in the host is being explored as a potential therapeutic strategy for VL. Hydrogen peroxide (H_2_O_2_), a potent ROS, induces a death mechanism in *L. donovani* promastigotes resembling metazoan apoptosis. H_2_O_2_ exposure reduces motility and survival, inducing apoptosis features like DNA fragmentation, which can be blocked by inhibiting caspase-like proteases. However, studies also show that cells infected with *L. donovani* fail to induce apoptosis after being treated with H_2_O_2_ due to their ROS-neutralising potential (Ref. 11). In *Leishmania*, ROS is also generated as a consequence of increased iron uptake, which occurs when the parasite encounters the iron-rich environment inside the macrophage. Iron plays a central role in many biochemical processes within the parasite, including energy production and the synthesis of essential macromolecules. However, the overabundance of iron can also lead to hydroxyl radicals (•OH), through the Fenton reaction leading to cellular damage (Ref. 12). Cells infected with *L. major* are also responsible for the generation of initial ROS (Ref. 13). Alternatively, reports from patients infected with *L. braziliensis* showed higher expression levels of ROS and NO in monocytes compared with healthy individuals; however, NO alone was not sufficient for parasite killing. Both ROS and NO were required for the control of the parasite (Ref. 14). In spite of having few detrimental roles such as killing parasites and activating the host immune response, ROS also triggers the differentiation of *Leishmania* sp. from the promastigote to the amastigote form. Studies on *L. amazonensis* have shown that ROS, particularly H_2_O_2_, act as a key signal for promastigote-to-amastigote differentiation, independent of pH or temperature changes. This transition occurs when mitochondria-generated superoxide radicals are converted into H_2_O_2_ by iron-dependent superoxide dismutase (SOD), either metabolically or by iron depletion in the culture medium. In *L. infantum*, exposure to elevated temperatures, which triggers differentiation, leads to mitochondrial hyperpolarisation, an increase in respiratory activity and a surge in ROS production. Promastigotes in the stationary growth phase can tolerate this ROS surge and successfully differentiate into amastigotes. However, log-phase promastigotes accumulate higher ROS levels, which leads to apoptosis but was reversed by over-expressing mitochondrial SOD (Ref. 15).

Another form of oxidative stress is reactive nitrogen species (RNS), which play a crucial role in the immune response against *Leishmania* by mediating parasite killing within activated macrophages. RNS, particularly nitric oxide (NO), are produced when inducible nitric oxide synthase (iNOS**)** is activated in response to interferon-γ (IFN-γ) and tumour necrosis factor-α (TNF-α) signalling. NO combines with superoxide to form peroxynitrite, a highly cytotoxic molecule that damages parasite DNA, proteins and membranes, leading to parasite clearance. In CL caused by *L. major*, a strong Th1 response promotes RNS production, contributing to parasite elimination. NO is considered essential in mice for controlling *L. major* infections, as it can diffuse across membranes to kill both intracellular and bystander parasites. iNOS deficient mice were susceptible to *L. major* infection even in the presence of strong T_H_1 responses compared to wild type (Ref. 13). *Leishmania* can secrete different peroxidases to neutralise the peroxynitrite (Ref. 16). Evidence suggests that pseudoperoxidase of *L. major* is also capable of detoxifying peroxynitrite (Ref. 17). In VL, persistent interleukin 10 (IL-10) and transforming growth factor-β suppress iNOS expression, reducing NO production and allowing parasite survival within macrophages, leading to chronic infection (Ref. 18). In DCL, amastigotes resist macrophage-mediated killing and grow intracellularly despite elevated NO synthesis compared to the promastigotes. This is due to the high expression of antioxidant enzyme tryparedoxin peroxidase (Ref. 19). In *L. braziliensis* infection, positive correlation was observed in the lesion size of the patients with the NO production (Ref. 14). Also, in *Leishmania-*infected macrophages, L-arginine is a key substrate for both NOS2 and ARG1, driving either parasite eradication via NO production or replication through polyamine synthesis. Amastigotes can metabolise L-arginine into polyamines or take them up from the host, enhancing replication. The roles of ROS are diverse, ranging from acting as signalling molecules in immune responses to playing a key part in pathogen defence and cellular regulation.

The following are the different roles of ROS.

### ROS in the induction of apoptosis

#### Cellular ROS

**Figure 2.**
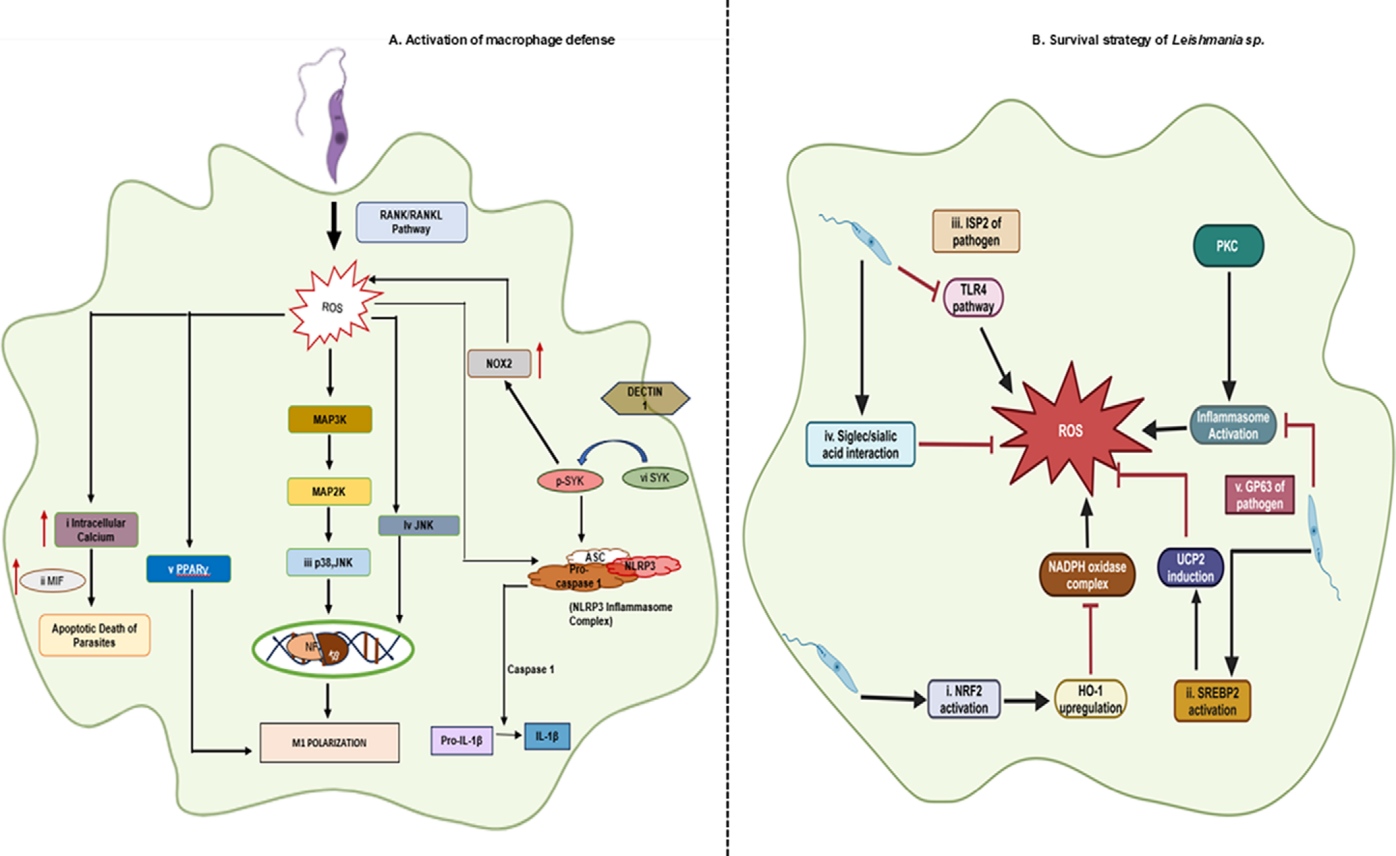
**A.** Effect of oxidative burst in macrophages following infection. During the initial phase of *Leishmania* infection, followed by its entry, macrophages produce transient ROS primarily through NOX2 activation and RANK/RANKL signalling. Various effects of ROS as a part of macrophage defence are summarised. **i.** ROS production causes enhancement in the cytosolic Ca^2+^ level, resulting in apoptotic death of the parasite. **ii.** Infection-induced ROS generation further upregulates MIF in macrophages, leading to parasite killing. **iii.** Oxidative stress induces proinflammatory cytokine production by activating p38/MAPK signalling cascade. **iv.** ROS activates JNK, which results in increased secretion of KC or CXCL1. **v.** ROS production facilitates M1 polarisation of macrophages via PPARγ activation. **vi.** Oxidative burst-dependent SyK activation further promotes the activation of NLRP3 inflammasome. **B**. Signalling pathways activated by *Leishmania* spp. to neutralise oxidative stress. In order to survive inside macrophages, *Leishmania* counteracts ROS production by: **i.** Infection-mediated activation of NRF2 induces the expression of a major antioxidant enzyme, HO-1. Infection-dependent dampening of NADPH oxidase assembly by HO-1 negatively regulates oxidative burst in macrophages. **ii.** Following infection, the production of UCP2, a negative regulator of ROS, gets upregulated through stabilisation and nuclear localisation of SREBP2. **iii.** *Leishmania* infection inhibits TLR signalling pathways ultimately diminishing ROS generation. **iv.** Interaction between siglec and sialic acid reduces ROS generation following phagocytosis of parasites by macrophages. **v.** Impairment of PKC activity minimises oxidative stress during infection.

Increased ROS generation following infection has been reported to play a crucial role in the induction of apoptosis of the parasite as well as of the host, thereby reducing infection (Ref. 20). ROS-mediated oxidative stress causes a significant increase in cytosolic Ca^2+^ level of the parasite, resulting in loss of mitochondrial membrane potential, which eventually leads to apoptosis-like death of *L. donovani* (Ref. 21) (Figure 2A(i)). Oxidative stress also occurs during *L. chagasi* infection, leading to lipid peroxidative damage in the liver (Ref. 22). Apart from VL, ROS is also associated with cell death in the case of CL. ROS results in endoplasmic reticulum (ER) stress-induced apoptosis in *L. major* through Ca^2+^-dependent mitochondrial toxicity (Ref. 23). Regarding host damage, in the context of *L. amazonensis* infection, ROS is also involved in parasite clearance. *L. amazonensis* infection leads to the upregulation of the NOX2 isoform of NADPH oxidase under hypoxic conditions, which further activates the transcription factor hypoxia-inducible factor 1-α (HIF-1α) through ROS generation. Here, the activation of the NOX2-ROS-HIF-1α pathway induces the production of macrophage migration inhibitory factor (MIF), which ultimately promotes the killing of *L. amazonensis* by macrophages (Ref. 24) (Figure 2A(ii)). Another study reported that NOX2-derived ROS also controlled *L. amazonensis* infection by facilitating neutrophil apoptosis and that inhibition of ROS aggravated disease pathology associated with loss of ear tissue (Ref. 25). *L. braziliensis* infection is also associated with oxidative burst. Monocytes from patients infected with *L. braziliensis* showed high levels of ROS expression, and in this case, ROS was also found to be intimately associated with parasite killing (Ref. 14).

#### Mitochondrial ROS

Mitochondria play a critical role in energy production and overall survival in *Leishmania* parasites. Oxidative stress, induced by ROS, can damage these organelles, particularly the inner mitochondrial membrane, which contains the kinetoplast. Damage to this membrane disrupts its structural integrity, potentially causing kinetoplast decondensation, which affects the parasite’s ability to maintain both its structure and function. Additionally, ROS-induced stress impairs the mitochondrion’s ability to sustain its membrane potential and ATP production, leading to a collapse of mitochondrial function. If this dysfunction is prolonged, it can ultimately result in the death of the parasite. K-09 treatment in *L. donovani* promastigotes induces mitochondrial dysfunction and ROS production, triggering apoptosis (Ref. 26). In *L. infantum*, mitochondrial superoxide leads to heat-induced apoptotic-like death, with early changes such as mitochondrial hyperpolarisation, increased respiration, and enhanced superoxide production, although cell death takes around 40 hours to complete (Ref. 27). In *L. amazonensis*, excessive ROS disrupt mitochondrial function, causing loss of membrane potential (ΔΨm), impairing ATP synthesis and releasing cytochrome c-like molecules, which trigger apoptosis (Ref. 28).

### ROS in immune activation

Successful parasite survival is known to be associated with a predominant anti-inflammatory environment within the macrophage, usually referred to as the M2 phenotype. On the contrary, interleukin 12 (IL-12) and TNF-α are produced by the host and contribute to the clearance of the parasite and are known to be M1 polarisation (Ref. 29). Another cytokine IFN-γ is a key driver of M1 macrophage polarisation in leishmaniasis, promoting parasite killing by enhancing ROS and RNS production, particularly NO. IFN-γ activates macrophages via the JAK-STAT1 pathway, upregulating pro-inflammatory cytokines like TNF-α and IL-12, which support Th1 responses. Research on C57BL/6 mice model has demonstrated that one of the reasons for their resistance against *Leishmania* infection is the maturation of the macrophages from M0 to M1 in the peritoneum. M1 macrophages exhibit high oxidative stress, which leads to the abrogation of the disease progression (Ref. 29). During VL, the generation of ROS activates p38 and ERK1/2/mitogen-activated protein kinase (MAPK) signalling pathways, resulting in an increased production of pro-inflammatory cytokines (Ref. 30) (Figure 2A(iii)), thereby producing a host favourable environment. In addition, in the case of CL, ROS is very closely associated with the activation of the host immune response. ROS-mediated activation of c-Jun N-terminal kinase (JNK) leads to increased secretion of keratinocyte-derived chemokine (KC) or chemokine (C-X-C motif) ligand 1 (CXCL1), a marker of inflammation in infected macrophages (Ref. 31) (Figure 2A(iv)). The receptor activator of nuclear factor-kappa B (RANK), a member of the TNF receptor family, activates various inflammatory signalling cascades, including nuclear factor-κB activation, inducible iNOS expression and subsequent NO production in macrophages. RANK-RANKL pathway provides protection against *L. major* infection, by shifting M2 macrophages into effector M1 type with a significant induction in NO and ROS production (Ref. 32). Similar to *L. major*, *L. mexicana* infection induces ROS production and polarises macrophage towards M1 profile via peroxisome proliferator-activated receptor γ (PPARγ) activation and subsequent downregulation of cytosolic phospholipase A2 and cyclooxygenase-2 (Ref. 33) (Figure 2A(v)).

### ROS in inflammasome activation

Interleukin-1β (IL-1β) is a proinflammatory cytokine that is involved in the restriction of infection during leishmaniasis. Maturation of pro-IL-1β into mature IL-1β is dependent on the activation of the NLR (nucleotide oligomerisation domain-like receptor) family pyrin domain-containing 3 (NLRP3) inflammasome complex consisting of NLRP3, ASC (apoptosis-associated speck-like protein containing CARD (caspase-associated recruitment domain)) adaptor and caspase-1. ROS is known to activate this NLRP3 complex. During *Leishmania* infection, immune cells like macrophages generate ROS in response to parasite recognition via pattern recognition receptors, leading to oxidative stress and mitochondrial dysfunction, which activate the NLRP3 inflammasome (Ref. 34). This triggers caspase-1 activation, processing pro-inflammatory cytokines such as IL-1β and IL-18, promoting inflammation and immune responses (Ref. 35). IL-1β plays a key role in controlling *Leishmania* infection by enhancing ROS/RNS production, driving a Th1 immune response, and upregulating iNOS for NO-mediated parasite killing. Inflammasome activation occurs in various forms of leishmaniasis, including CL, VL and MCL, contributing to both immune defence and pathology (Ref. 36). In CL, IL-1β can influence disease progression or resolution, with increased NLRP3 expression linked to *L. braziliensis* pathology (Refs 37, 38) and *L. major* (Ref. 39). Dysregulated IL-1β in VL, caused by *L. donovani*, leads to excessive inflammation and pathology (Ref. 40). On the other hand, reports have shown that *L. donovani* may evade IL-1β-mediated immunity by inducing IL-10, which dampens inflammation and aids parasite persistence. Macrophages infected with *L. amazonensis* also facilitate inflammasome activation and parasite killing via inducible NO (Ref. 37). Inflammasome activation varies across different cell types, including neutrophils, dendritic cells and keratinocytes. In *L. amazonensis* infection, Mon-DCs show poor inflammasome activation (Ref. 41), while keratinocytes infected with *L. infantum* exhibit increased pro-inflammatory gene expression, including TNF-α and IL-1β (Ref. 42). ROS production in macrophages, neutrophils and keratinocytes activates the inflammasome, primarily via Dectin-1 and Spleen Tyrosine Kinase (SyK) (Ref. 43) (Figure 2A(vi)). Clec7a−/− mice treated with *L. amazonensis* showed failure in inhibiting parasite replication, highlighting the role of Dectin-1-induced SyK activation in inflammasome assembly and reducing parasite persistence (Ref. 43). Thus, IL-1β is a crucial regulator of host defence, balancing inflammation and parasite restriction to influence disease outcomes.

## Neutralisation of host oxidative stress by the parasite: Strategies for survival

In order to successfully survive within the hostile environment of host cells, *Leishmania* also employs efficient strategies to dampen the host antimicrobial arsenals (Ref. 8). As discussed earlier, immediately after the phagocytosis of the pathogen, the first line of defence put forward by the host is oxidative burst (Ref. 6). For its intracellular survival and establishment of infection, the parasite exploits various host signalling mechanism in order to successfully combat the host oxidative burst.

### Activation of host antioxidant enzymes

In the case of VL, upon infection, there is a marked upregulation of the antioxidant enzyme heme oxygenase-1 (HO-1). HO-1 induction results in the depletion of cellular haem level, thereby blocking the haem-dependent maturation of gp91phox, a major component of the principle ROS-producing enzyme NAD(P)H oxidase. The absence of haem blocks the maturation of gp91phox, which ultimately prevents the assembly of the NAD(P)H oxidase enzyme complex, a key enzyme for ROS production (Ref. 6). This enhancement of HO-1 expression is mainly regulated by the transcription factor nuclear factor erythroid 2-related factor (NRF2). Infection-induced production of ROS results in the dissociation of NRF2 from its inhibitor Kelch-like ECH-associated protein 1 and facilitates the nuclear translocation of NRF2 (Ref. 44) (Figure 2B(i)). In addition to *L. donovani*, induction of HO-1 metabolism is also observed in dogs infected with *L. infantum*, and this may be responsible for the reduction in the oxidative and nitrosative metabolism of macrophages (Ref. 45). HO-1 also escalates the survival of *L. chagasi* within macrophages by repelling inflammation and oxidative stress. Induction of HO-1 inhibits the production of ROS and some major pro-inflammatory cytokines such as TNF-α and interleukin 6 (IL-6) (Ref. 46). *Leishmania pifanoi* amastigotes also diminish superoxide production by inducing haem degradation through a significant upregulation in HO-1 expression (Ref. 47). Treatment of primary human macrophages with Resolvin D1, a key eicosanoid known for its anti-inflammatory and pro-resolving effects, enhances the intracellular replication of *L. amazonensis.* This effect appears to be linked to the induction of HO-1, a protein involved in regulating inflammation and cellular stress responses (Ref. 48).

### Induction of key regulatory proteins within the host

Apart from cellular ROS, mitochondrial ROS (mt-ROS) is also regulated by *Leishmania. L. donovani* infection is associated with a significant upregulation of uncoupling protein 2 (UCP2), a negative regulator of mt-ROS generation (Ref. 30). UCP2-mediated ROS inhibition further inhibits the production of a major pro-inflammatory cytokine IL-1β (Ref. 40). *L. donovani* infection leads to the activation of the Lyn (tyrosine-protein kinase)/PI3K (phosphoinositide 3-kinase)/Akt pathway, which, in turn, facilitates the stabilisation and nuclear translocation of sterol regulatory element binding protein 2 (SREBP2). The activation of SREBP2 transcriptionally upregulates UCP2 production following infection (Ref. 49) (Figure 2B(ii)). Moreover, *L. donovani* also inhibits oxidative stress-mediated apoptosis of macrophages by inducing suppressors of cytokine signalling (SOCS) proteins. The expression of both SOCS1 and SOCS3 proteins were significantly induced in *L. donovani*-infected macrophages (Ref. 11). Similar to VL, the parasite also inhibits the generation of ROS in the case of CL to facilitate its survival inside the host cell. The inhibitor of serine peptidase 2 of *L. major* prevents the activation of Toll-like receptor 4 (TLR4)–neutrophil elastase signalling cascade during the early phase of the parasite–macrophage interaction. Although this signalling pathway leads to the downregulation of parasite phagocytosis by macrophages, it also exerts a beneficial effect on intracellular parasite survival due to the inhibition of ROS generation (Ref. 50) (Figure 2B(iii)).

### Involvement of surface molecules of Leishmania: Protectors of parasite

Lipophosphoglycan, a surface molecule present on the membrane of *L. donovani* promastigotes, has been found to be responsible for the reduction of superoxide radical production by disrupting the assembly of functional phagosomal NAD(P)H oxidase complex. Here, infection interferes with the recruitment of the cytosolic components p47phox and p67phox of NAD(P)H oxidase at the phagosomal membrane (Ref. 51) (Figure 2B(i)). Moreover, *L. donovani* uses sialic acids for the downregulation of ROS production. Sialic acid-binding immunoglobulin-like lectins (Siglecs) are one type of receptor present on the membranes of haematopoietic cell lineages that interact with linkage-specific sialic acids. During phagocytosis, the interaction between siglec and sialic acid dampens ROS generation, inducing enhanced multiplication of amastigotes within the host environment (Ref. 52) (Figure 2B(iv)). Similar to *L. major*, GP63, a surface molecule of *L. mexicana*, leads to the inhibition of macrophage IL-1β production. This parasite-mediated suppression of IL-1β secretion is associated with the inhibition of ROS production, which further inhibits the activation of NLRP3 inflammasome. The reduction in the level of ROS production is due to the impairment of protein kinase C (PKC)-mediated protein phosphorylation (Ref. 53) (Figure 2B(v)).

## Oxidative stress-induced changes within *Leishmania* sp.: An attempt to adapt and evolve

Apart from manipulating host activation pathways, some of the genes within *Leishmania* sp. also help themselves to overcome the hurdle of oxidative stress. These genes may be associated with antioxidant systems, metabolic pathways, DNA replication machinery and a few other signalling cascades.

### Key regulatory enzymes of Leishmania in subversion of oxidative stress

At the time of phagocytosis by macrophages, the parasites are exposed to a higher temperature (37°–38°) from a relatively lower temperature (25°–27°). This sudden change in temperature induces some major changes within the parasite, including an increased cellular respiration rate associated with high mitochondrial superoxide production (Ref. 27). SOD is an important antioxidant defence enzyme in the case of parasite survival for its role in the detoxification of superoxide into H_2_O_2_ and oxygen. As previously mentioned, during the course of *L. donovani* infection, mitochondria produce huge amounts of superoxide radicals as a result of cellular respiration, and therefore targeting of SOD to the mitochondria is indispensable for its proper activity. Iron superoxide dismutase (Fe-SOD) A is thus targeted to mitochondria by an N-terminal 31 amino acid pre-sequence (Ref. 54). Secretion of Fe-SOD by promastigotes of *L. infantum* also plays a pivotal role in ROS neutralisation during the course of infection (Ref. 55). SOD, especially SODB1 of *L. major*, helps in antioxidant defence by converting superoxide to oxygen and H_2_O_2_; hence, the deletion of this gene impairs the survival of the pathogen (Ref. 56).

Glutathione peroxidase (GPx) is crucial for the survival of *Leishmania* parasites by protecting them from oxidative stress induced by the host’s immune system, particularly in CL caused by species such as *L. major* and *L. tropica*, where it helps the parasite survive within skin macrophages (Ref. 57). Trypanothione reductase (TR) is also vital for parasite survival, as it reduces trypanothione, a key molecule in the parasite’s antioxidant defence. This reduced trypanothione neutralises ROS, especially H_2_O_2_, produced by host macrophages during the immune response. The tryparedoxin/tryparedoxin peroxidase system further uses reduced trypanothione to protect the parasite from oxidative damage. TR is essential for protecting *L. donovani* from metalloid-induced oxidative stress (Ref. 58). Studies on *L. infantum* have shown that cyclobenzaprine induces ROS, inhibits TR and reduces the parasite load (Ref. 59).

The ROS-directed activation of soluble adenylate cyclase of *L. donovani* regulates the cyclic AMP-protein kinase A signalling cascade for its survival within the host macrophage during infection (Ref. 60). In response to oxidative stress, the expression of the mevalonate kinase (*MVK*) gene increases within *L. donovani*, which causes significant enhancement in their ergosterol content. This elevated ergosterol level provides oxidative stress tolerance to the pathogen by inhibiting peroxidative degradation of cellular lipids and conserving plasma membrane integrity (Ref. 61). Oxidative stress also induces the expression of uracil DNA glycosylase in *L. donovani*, which provides protection against some ROS-inducing antileishmanial drugs (Ref. 62). Oxidative stress upregulates the expression of another enzyme DNA polymerase β in *L. donovani*, and this enzyme, in turn, counteracts oxidative stress by reducing the intracellular level of ROS (Ref. 63). Arginine succinate synthase of *L. donovani* is also involved in parasite survival. (Ref. 64). DNA polymerase θ, a translesion synthesis polymerase of *L. infantum*, also facilitates parasite viability by inducing tolerance against macrophage-generated oxidative damage (Ref. 65). Gamma-glutamyl cysteine synthetase, which is encoded by the *GSH1* gene, is the rate-limiting enzyme in glutathione and trypanothione biosynthesis in *Leishmania.* The lack of the GSH1 allele increases the susceptibility of *L. infantum* towards oxidative stress, thus indicating its role in controlling oxidative stress management (Ref. 66).

In addition to VL, some genes within parasites are also involved in counteracting oxidative bursts during CL. Ascorbate peroxidase (APX) of *L. major* is associated with the neutralisation of oxidative stress. Overexpression of this enzyme hampers mt-ROS production and provides protection against mitochondrial cardiolipin oxidation to the parasite. This enzyme also inhibits oxidative stress-directed programmed cell death of *L. major* (Ref. 67). Therefore, APX is one of the essential factors required for the intracellular survival of the parasite (Ref. 68). Pteridine reductase 1 (PTR1) of *L. major* also protects the parasite against oxidative stress. The depletion of PTR1 increases the sensitivity of the parasite towards H_2_O_2_, whereas the overexpression of PTR1 leads to significant enhancement in oxidant resistance (Ref. 69). The lack of NAD(P)H cytochrome b5 oxidoreductase in *L. major* also results in increased oxidative stress-mediated death of the parasite through the impairment of linoleate synthesis (Ref. 70). Increased expression of cysteine synthase and cystathionine β-synthase facilitates the survival of *L. braziliensis* under oxidative stress (Ref. 71).

### Transcriptional and translational modulation within Leishmania spp. to neutralise ROS

DDX3, a conserved ATP-dependent DEAD-box RNA helicase, plays a central role in mitochondrial protein quality control in *L. infantum*, protecting the parasite from ROS-induced damage (Ref. 72). In *L. donovani*, after macrophage phagocytosis, oxidative burst triggers phosphorylation of translation initiation factor eIF2α via PERK, reducing global translation and promoting redox homeostasis genes for parasite persistence (Ref. 73). The *Atg8* gene in *L. donovani* is crucial for intracellular survival and regulates differentiation to amastigotes, impacting infectivity (Ref. 74). The amastigote-specific A2 protein protects *L. donovani* against oxidative stress but not ER stress (Ref. 75). In *L. chagasi*, increased host temperature and oxidative stress trigger the synthesis of heat shock protein 70 (HSP70), which helps resist oxidants, and *L. amazonensis* HSP70 also counters oxidative stress (Ref. 76). LABCG1 and LABCG2, ATP-binding cassette transporters in *L. major*, are critical for infectivity and virulence; their deficiency increases ROS production, highlighting their role in oxidative stress control (Ref. 77).

### ROS in parasite metabolism

*Leishmania* species adapt their metabolism to various environments, such as the sandfly vector and host macrophages, with ROS production playing a central role. In the promastigote stage, glycolysis converts glucose to pyruvate, generating ATP and NADH, while the pentose phosphate pathway (PPP) produces NADPH to neutralise ROS and maintain redox balance. *L. donovani* relies on glycolysis and PPP to manage oxidative stress during host invasion and replication (Ref. 78). *Leishmania* competes with macrophages for iron, but to avoid iron-induced ROS production, the parasite manipulates macrophage iron trafficking, enhancing iron availability through the fusion of phagolysosome and endocytic vesicles containing transferrin-bound iron like in the case of *L. amazonensis* infection (Refs 79, 80). Additionally, *Leishmania* species suppress the expression of ferroportin, preventing iron export and increasing its availability for the parasite (Ref. 81). Arginine metabolism is also crucial in *Leishmania* infection, with parasite growth and persistence linked to increased host Arginase 1 activity. In *L. amazonensis*-infected macrophages, metabolomic analysis shows enhanced L-arginine metabolism towards polyamine production, which improves the redox balance of infected cells, protecting the parasites from host-derived NO and ROS (Ref. 82). In the promastigote form, *Leishmania* uses mitochondria for oxidative phosphorylation, which generates ROS as a byproduct of the electron transport chain. *L. donovani* induces mitochondrial UCP2 to reduce mt-ROS levels, aiding its proliferation within host macrophages. Knockdown of UCP2 leads to higher mt-ROS levels and a reduced parasitic load. UCP2, in combination with A-20, an NF-κB inhibitor, suppresses NLRP3 inflammasome activation, promoting disease progression. This mechanism links UCP2 to metabolic regulation, as it helps the parasite balance oxidative stress and enhance its survival in the host (Ref. 83). Fatty acid metabolism is another key aspect of *Leishmania* metabolism. In the amastigote form, *Leishmania* relies on fatty acid β-oxidation in peroxisomes to generate ATP (Ref. 84). The oxidation of fatty acids produces H_2_O_2_, a form of ROS, which is further metabolised by the parasite’s antioxidant systems. APX is a redox enzyme involved in the trypanothione pathway, responsible for converting H_2_O_2_ into water (Ref. 85).

## ROS activation: Targets of potential antileishmanial drugs and natural compounds

Several drugs exert their antileishmanial activity by inducing ROS production. Miltefosine, a potential anticancer drug, causes ROS-mediated depletion in parasitic load in the case of *L. donovani* infection by impairing the NRF2/HO-1 axis (Ref. 86). Cyclobenzaprine, a well-known muscle relaxant, raises ROS levels in *L. infantum* and reduces parasite load in infected mice (Ref. 59). Imidazole drugs such as clotrimazole, econazole and bifonazole also show antileishmanial activity with a significant induction in ROS generation within *L. infantum* (Ref. 87). Novel organic compound 1,2,3-triazole derivatives also promote mitochondrial-ROS production and depolarisation of mitochondrial membrane potential of *L. amazonensis* (Ref. 88). In addition to this, few natural compounds are also involved in the killing of *Leishmania* sp. without causing significant damage to the host cell. Oleuropein, a biophenol derived from the olive tree, exhibits leishmanicidal effects by inducing ROS production both in the cases of *in vitro* and *in vivo* models of *L. donovani* infection (Ref. 89). Sesamol (3,4-methylenedioxyphenol) inhibits growth and proliferation of *L. donovani* promastigotes in a dose-dependent manner. In addition, it also reduces the intracellular parasite load during infection. This compound shows its antileishmanial effects by inducing ROS, which ultimately kills the parasite (Ref. 90). Another natural compound, betulin, stimulates ROS production, which eventually causes apoptosis-like death of *L. donovani* (Ref. 91). Some natural sesquiterpene lactones also induce oxidative stress in *L. mexicana* (Ref. 92). An alkaloid berberine chloride has been also studied to enhance the oxidative burst and decrease the parasite persistence of *L. donovani* inside the cells through the induction of apoptotic-like death (Ref. 93).

## Concluding remarks

Leishmaniasis is a neglected tropical disease, caused by the eukaryotic parasite, *Leishmania.* The production of ROS is one of the major host defence mechanisms to eliminate intracellular pathogens, but surprisingly *Leishmania* manages to thwart this security system needed for establishing infection. The process is not always a smooth sail, as the host also tries its best to resist the parasite’s effort. ROS generation leads to the activation of several host signalling pathways, which further enrich the immune environment of the host. In contrast, parasite exploits negative regulators of the host pathway to deactivate the immune system and ultimately it becomes the ‘survival of the fittest’. The survival of *Leishmania* species is inversely related to oxidative stress, as increased levels of ROS can damage the parasite’s cellular components, potentially leading to its death. However, *Leishmania* has evolved several defence mechanisms, such as antioxidant enzymes and specialised organelles like peroxisomes, to neutralise ROS and counteract oxidative damage. These adaptive survival strategies help the parasite to control a balanced redox state, allowing it to survive despite the host’s immune response, which generates oxidative stress to kill the parasite. Thus, while oxidative stress poses a threat to *Leishmania*, the parasite, on the other hand, tries to evade the immune defence. This inverse relationship between oxidative stress and parasite survival means that increased stress typically reduces survival unless the parasite activates its defence mechanisms. Its survival depends on the ability to effectively manage and mitigate this stress (Ref. 94).

Exploiting ROS against the weak defence system of *Leishmania* may involve strategies that overwhelm its antioxidant defences, making it vulnerable to oxidative damage. *Leishmania* parasites rely heavily on their antioxidant enzymes, such as TR, SOD and GPx, to neutralise ROS produced by the host’s immune response. By inhibiting these antioxidant pathways, either through drug-based inhibition or through genetic knockdown, we can impair the parasite’s ability to detoxify ROS, thereby enhancing oxidative stress within the parasite. Additionally, boosting the host’s ROS production through the activation of immune cells, like macrophages, can create an inhospitable environment for *Leishmania.* Stimulating macrophages to produce higher levels of ROS, via activating NADPH oxidase or iNOS, further damages the parasite. Targeting *Leishmania* with pharmacological agents that directly generate ROS or disrupt its mitochondrial function is another promising strategy. Certain drugs can induce mitochondrial dysfunction or ROS production within the parasite, overwhelming its defences and leading to cell death. Additionally, the use of nanoparticles designed to deliver ROS-generating compounds directly to *Leishmania*-infected macrophages can localise oxidative damage, sparing surrounding healthy cells and enhancing the host’s immune response. Finally, inhibiting *Leishmania*’s ROS-detoxifying pathways, like the trypanothione system, can tip the balance in favour of oxidative stress, further weakening the parasite’s survival. Many laboratories, including ours, are trying to identify the detailed signalling pathway behind the regulation of ROS and its modulation by the pathogen for the development of a more effective therapeutic approach.
